# Users’ Opinion About a Virtual Reality System as an Adjunct to Psychological Treatment for Stress-Related Disorders: A Quantitative and Qualitative Mixed-Methods Study

**DOI:** 10.3389/fpsyg.2018.01038

**Published:** 2018-06-22

**Authors:** Verónica Guillén, Rosa M. Baños, Cristina Botella

**Affiliations:** ^1^Department of Personality Evaluation and Psychological Treatment, University of Valencia, Valencia, Spain; ^2^CIBER of Physiopathology of Obesity and Nutrition, Madrid, Spain; ^3^Department of Basic Psychology, Clinical and Psychobiology, Jaume I University, Castellón de la Plana, Spain

**Keywords:** virtual reality, psychological treatments, expectations, opinion, stress related disorders

## Abstract

This study aims to explore patients’ and therapists’ attitudes about the psychological treatment they received (patients) or applied (therapists). The treatments were standard CBT protocols for post-traumatic stress disorder (PTSD), complicated grief (CG), or adjustment disorders (ADs), depending on each patient diagnosis. The treatments were delivered following a traditional format or supported by a virtual reality (VR) system “EMMA’s WORLD” designed for the treatment of stress-related disorders. “EMMA’s WORLD” is a VR application in which patients can explore negative experiences using different virtual elements that can be customized to make them more meaningful to the user. The sample was composed of two groups: the “professionals” (*N* = 10) were all clinical psychologists who applied the same psychological treatment in both the traditional format (“traditional condition”) and using the VR system (“*EMMA”* condition). The second group consisted of a sample of patients (*N* = 50) who met the criteria for at least one of three different diagnoses: PTSD (*N* = 15), CG (*N* = 15), or AD (*N* = 20). 25 patients received treatment in the traditional format and 25 supported by the VR system. The patients were asked about their expectations (before treatment) and satisfaction (after treatment) with the treatment they received. All the therapists were asked their opinions about both treatment conditions. A mixed-methods approach using quantitative and qualitative methodologies was used. In both conditions, high scores were observed, and the patient’s opinions were even better when they have already received the treatments. A more pronounced pre-test–post-test change in the EMMA therapy group than in the traditional group was observed. *EMMA’s World* was well-accepted by both patients and therapists, and it helped to foster motivation in patients, while helping the therapist to apply the treatment. Thus, VR can be useful as an adjunct tool to enhance the treatment.

## Introduction

In the DSM-5, disorders that are precipitated by specific stressful and potentially traumatic events are included in a new diagnostic category, “trauma and stressor-related disorders" (SRDs) (American Psychiatric Association [APA], 2013). These disorders occur when the person is not able to cope with traumatic events or stressful situations and, consequently develops a series of clinical symptoms that can significantly affect his or her life. SRDs include several disorders and from a clinical point of view, it is useful to group these disorders together in order to distinguish, between different degrees of psychopathological severity, ranging from normal (non-pathological) distress, through clinical acute elevated stress, as in adjustment disorders (ADs), to more serious and severe psychopathology, as in post-traumatic stress disorder (PTSD) (Friedman et al., 2011).

Fortunately, we now have excellent evidence based psychological treatment strategies that have shown their effectiveness. *In vivo* exposure therapy is considered one of the most effective treatments for many psychological disorders including SRD (Barlow et al., 2015). A significant empirical base supports the efficacy of *in vivo* exposure, and data from meta-analyses consistently show its usefulness (Deacon and Abramowitz, 2004). Cognitive Behavior Therapy programs that include exposure-based techniques have been shown to be effective for the treatment of SRDs (Najavits and Anderson, 2015). Without a doubt, exposure therapy can be considered one of the great success stories within the field of mental health (Olatunji et al., 2009). Nevertheless, exposure therapy is under-utilized in clinical settings (Becker et al., 2004). The data indicate that, when they realize that the exposure involves direct confrontation with the feared object or context, approximately 25% of patients reject or abandon it after starting (Marks and O’Sullivan, 1992; Choy et al., 2007; García-Palacios et al., 2007; Ong et al., 2016) because it is too aversive for them. Likewise, many therapists have difficulties or feel reluctant when using exposure with their patients because it might lead to a worsening of patients’ symptoms or treatment rejection (Feeny et al., 2003; Becker et al., 2004; Richard and Gloster, 2007; Deacon et al., 2012). For example, Becker et al. (2004) found that in a sample of 852 well-trained psychologists, only 17% of the clinicians used exposure to treat PTSD. Along the same lines, Richard and Gloster (2007) surveyed clinicians and found that exposure is considered a quite aversive treatment. All this indicates that some professionals maintain a negative view of this technique (Olatunji et al., 2009).

Virtual reality (VR) can contribute to improving the clinical utility of exposure therapy, by making it more easily accepted (Botella et al., 1998). VR refers to the three-dimensional digital worlds where the person can navigate and interact in real time, even though these worlds only exist in the memory of the computer. In the first case study conducted, VR was used with an acrophobic patient to apply virtual exposure to her feared situations (Rothbaum et al., 1995). This pioneering work raised expectations about the utility of this technology in employing the exposure technique. Basically, the exposure is designed in such a way that the patient gradually and systematically faces the situations, objects, or activities that he/she fears and avoids.

The new VR generation offers new opportunities for the presentation of stimuli and contexts in ways that are impossible in traditional *in vivo* exposure therapy (Lindner et al., 2017). In the past 15 years, the use of VR as an exposure technique has increased in terms of the number of problems it addresses and their complexity, and review studies and meta-analyses have been published on this topic (Powers and Emmelkamp, 2008; Gerardi et al., 2010; Meyerbröker and Emmelkamp, 2010; Opris et al., 2012; McCann et al., 2014; Turner and Casey, 2014; Botella et al., 2015, 2017; Morina et al., 2015). The results of all these studies show that VR is a valuable tool that helps to carry out the exposure. VR allows a high level of control by both the therapist and the patient, offering the possibility for a very accurate gradation of the exposure tasks and making it possible to design the virtual exposure treatment to fit each patient’s needs. The context generated by the computer offers a protected environment where the person can cautiously face the feared situation at his/her own pace and speed. Therefore, VR can be a key intermediate step between the therapist’s consulting room, which is completely protected, and the threatening real environment. VR allows the feared environments to be customized according to the needs and preferences of users, who can repeat the exposure several times until the fears are overcome. Moreover, the feared situation is not always easily accessible (e.g., a cave, a tunnel). In these cases, imagery exposure is usually used, but this technique is less effective than *in vivo* exposure, and there are individual differences in imaginative ability (Botella et al., 1998). VR provides more realistic stimuli for people who have trouble imagining. Furthermore, VR makes it possible to go beyond reality and create situations or elements that are so threatening that they are not likely to occur in real life (e.g., in the virtual world we have developed for claustrophobia, a wall can move forward, making a loud noise and leaving the patient enclosed in a small space). Finally, VR is useful from an ethical point of view; it protects the patient’s privacy and intimacy because it is not necessary to leave the office to perform the exposure tasks.

Despite these advantages, there are also some problems and barriers associated with the use of VR. According to Riva (2005), these barriers include the lack of standardized clinical protocols for applying VR shared by the scientific community; the final costs of the programs, although these costs actually decrease dramatically from year to year; or ethical and security issues regarding to possible side effects (Durlach and Mayor, 1995; Riva, 2005). However, it is also true that most people who use VR systems either do not manifest any problem associated with the use of this technology, or they experience mild and transient symptoms such as dizziness and discomfort (Nichols and Patel, 2002). Finally, there are other barriers that impede the use of VR in everyday clinical practice. On the one hand, some clinicians find it difficult to accept the use of technologies because they feel threatened and nervous about working with them. On the other hand, some clinicians are reticent about the effect the use of these technologies may have on the therapeutic relationship, although studies have shown that this is not a problem (Andersson, 2009; Wrzesien et al., 2012, 2013). In this regard, it should be noted that the purpose of VR and other applications based on technological advances is simply to improve the treatment, and not to make the therapist’s work superfluous or less meaningful. One study (Segal et al., 2011) analyzed therapists’ perceptions of the benefits and associated costs of the use of VR in the field of psychological treatments. A sample of 271 professionals completed an online survey, and the results indicated that overall they perceived that the potential benefits significantly exceeded the potential costs. However, this study only provides information about the therapists’ intentions to use or not use VR because the therapists did not actually use VR. In addition, data were obtained from a set of pre-selected items, and no information was offered about therapists’ opinions about positive and negative issues from their real experience using VR. In fact, only 3% of the therapists who participated in the study had experience in the current use of this technology when applying psychological treatments.

In sum, VR has been shown to be an effective technique that can be useful to reduce the percentages of patients’ and therapists’ rejection of the exposure technique. For this reason, it is necessary to study the opinions of the “users,” both therapists and patients, about the different ways of applying this exposure technique, and find out to what extent they think tools like VR enhance or complement traditional treatments, further complicate the treatment, or can even be a disturbing element in the therapeutic process.

Our group has developed a VR system called “EMMA’s World” a VR system specifically designed for the treatment of PTSD, CG, and AD to facilitate emotional change in people who have suffered traumatic or stressful experiences (see Rey et al., 2005; Botella et al., 2006, for a more detailed description). In *EMMA’s World* patients can explore traumatic or stressful experiences to the degree required for specific therapeutic needs. The system shows customized, clinically significant environments for each patient, emphasizing the meaning of the trauma or stressful event over the realism of the VR environment. The efficacy of this system has been shown in treating SRD, and these results were maintained at follow-ups (Baños et al., 2009; Botella et al., 2010). Furthermore, it has been compared to traditional treatments (Baños et al., 2011), revealing that both treatment programs (traditional and VR) were effective for these problems. However, when differences were found between them, they favored the protocol supported by EMMA’s World, especially when the results are considered in the medium term (6-month follow-up). In sum, EMMA’s World has been shown to be an effective and versatile device. However, regardless of the efficacy data, the literature suggests that there may be differences between treatments preferred by therapists and those preferred by patients. Thus, further research is needed to better understand their attitudes and opinions about exposure therapy and help to explain the underutilization of this treatment in clinical practice (Becker et al., 2007).

The objective of this study is to analyze patients’ and therapists’ opinions about EMMA’s World, used as an adjunct tool to improve the efficiency (understood as acceptability) of evidence-based psychological treatment protocols for the treatment of SRD. We will explore the opinions of patients and therapists about the potential benefits and drawbacks of using (or not) EMMA’s World. In other words, the purpose is to find out their views about the psychological treatment they received (patients) or applied (therapists) following a traditional format, or supported by EMMA’s World. We want to discover whether EMMA’s World has been useful or has, in some way disturbed the process of change, and what aspects of this system could be improved. Based on the literature on using VR for other psychological problems (Botella et al., 2007; Baños et al., 2009; McCann et al., 2014; Freeman et al., 2017), we expect the VR system to be considered useful for therapy and well-accepted by both patients and therapists.

## Materials and Methods

### Participants

The study sample consisted of two groups. The first group was composed of “professionals” (*n* = 10), clinical psychologists who applied the treatment in a traditional format (“traditional” condition) and the same treatment protocol using the VR system (“EMMA” condition; see description below). All the professionals had been applying cognitive behavioral therapy for SRD in clinical practice for more than 7 years. All of them had at least a master’s degree in clinical psychology, and the majority had a Ph.D. They all worked at the PREVI Clinical Psychology Center or at the Emotional Disorders Clinic at Jaume I University (Spain). The treatment protocols had been manualized to ensure their homogeneity, and a senior clinician supervised the application of the treatments. Moreover, all the professionals were experts in the use of technology, specifically VR, for delivering psychological treatments.

The second group was composed of a convenience sample of patients (*n* = 50), who met the criteria for at least one of three different diagnoses according to the DSM-IV (American Psychiatric Association [APA], 1994): PTSD (*n* = 15), CG (*n* = 15), or AD (*n* = 20). They had participated in two previous studies conducted by our team comparing the efficacy of the same psychological treatment protocol with and without EMMA’S World (two doctoral dissertations: Andreu-Mateu, 2011; *N* = 46 and Guillén, 2008; *N* = 39). In these two studies, the same method of randomization was used. Participants were randomly assigned to one of the two treatment conditions: “traditional condition” and “EMMA condition” using a computer-generated random number sequence. The Epidat 4.1 program was used to generate this sequence. Randomization was stratified by primary diagnosis. Block randomization was performed within each stratum in order to ensure all primary diagnoses were equally represented in both experimental conditions. The allocation was carried out by an independent person who was not involved in the study. The allocation sequence was concealed until interventions were assigned. These two studies have been approved by the Ethical Committee of Jaume I University.

The inclusion criteria for all the cases were: (1) meeting the criteria for one of the three diagnoses (PTSD, CG, or AD); age (2) between 18 and 65 years; and (3) in the case of taking medication, not increasing the dose or changing the medication during the study; (4) and not being presently involved in other psychological treatment. The exclusion criteria were: (1) a diagnosis of psychosis diagnosis or severe personality disorder; (2) suffering from a severe and incapacitating physical illness; (3) substance dependence or abuse; and (4) secondary gains derived from the problem (e.g., work leave, work disability, etc.). In the present study were included the participants who: (1) had completed the treatment in the previous study and were therefore able to give their opinion about the treatment received; and (2) answered the evaluation questionnaires and gave their opinion about the treatment received.

All participants signed the informed consent before starting the treatment. Patients were treated at the PREVI Clinical Psychology Center or the Emotional Disorders Clinic at Jaume I University (Spain). In the latter case, they were recruited through derivations from Mental Health Units and announcements posted at Jaume I University, the University of Valencia, and the Polytechnic University of Valencia. The announcements, offered psychological treatment to overcome stressful and traumatic events.

### Measures

#### Clinician-Administered PTSD Scale (CAPS; Blake et al., 1990, 1995)

This interview rates whether there have been traumatic events in the person’s life and the symptomatology associated with these events, and it makes it possible to establish the diagnosis of PTSD according to the DSM-IV. The CAPS allows the evaluator to make an overall assessment of the severity of the PTSD, the improvement since the prior assessment, and the validity of the responses. Different studies have shown that it has high reliability (internal consistency and inter-rater reliability and convergent validity (Blake et al., 1990, 1995; King et al., 1998). Results have also been reported about the sensitivity of the treatment (Thompson et al., 1995). The mean score on the CAPS in a clinical sample was 45.9 (*SD* = 29.1) (Blanchard et al., 1996).

#### Inventory of Complicated Grief (ICG) (Prigerson et al., 1995, 1999)

This inventory is composed of 19 items to be rated on a five-point scale from 0 (“never”) to 4 (“always”). This instrument assesses the level of the person’s adjustment to grief or its interference in his or her life. Exploratory analyses of the inventory have shown high internal consistency and test–retest reliability, as well as high convergent and criterion validity. In addition, an association has been found between the scores on the ICG and the severity of depressive symptoms, along with other measures of emotional distress. Therefore, it is an adequate instrument to assess complicated grief (CG) symptoms (Prigerson et al., 1995, 1999).

#### Diagnostic Interview for Adjustment Disorders

It is a semi-structured interview developed by our research group to assess AD, taking into account the data provided by the scientific literature, the diagnostic criteria of the ICD-10 and the DSM-IV-TR, and the SCID-IV interview (First et al., 2002). It also includes a list of 28 symptoms related to AD whose presence and severity can be rated on a Likert scale from 0 (“not at all”) to 8 (“very serious”). It has been applied in other studies (Andreu-Mateu, 2011; Andreu-Mateu et al., 2012).

#### ‘Treatment Expectations Scale’ and ‘Treatment Satisfaction Scale’

Are an adaptation of Borkovec and Nau (1972) instrument for assessing the patients expectations before starting the treatment (once it has been explained), and their opinion and satisfaction about the treatment after finishing it. Each scale consists of five items, rated from 0 (‘strongly disagree’) to 10 (‘strongly agree’), covering different domains related to the treatment: logicalness, credibility/satisfaction, success in reducing symptoms, usefulness for the patient’s specific problem, usefulness for treating other psychological problems, confidence in recommending it to other people with the same problem, and unpleasantness/aversiveness. The expectations scale is applied once the treatment rationale has been explained in the second assessment session. Its aim is to measure the participant’s subjective expectations about the intervention. The satisfaction scale is administered when the participant has received the treatment, and its aim is to assess the patient’s opinion of the intervention. We have used this instrument in previous studies (Botella et al., 2008, 2009, 2016).

#### Opinion Sheet on Treatment

Once the treatment was finished, both patients and therapists were asked to freely indicate their opinions about the treatment they had received (patients) or applied (therapists). They were explicitly asked to assess to what extent the treatment had been useful for them (or not) in achieving their therapeutic objectives. No restrictions were placed on length, and so each participant was free to write as much as he/she desired. Every patient could assess the treatment received, whether it was the treatment protocol following traditional therapy, or the same protocol supported by VR EMMA’s World. In the case of the therapists, they were asked to give their opinions about both application protocols. All participants were also asked to indicate anything related to the treatment received (or applied) that could be useful for future improvements.

### The Virtual Environment: EMMA’s World

The VR system called EMMA’s World was developed to achieve two fundamental objectives in clinical practice: on the one hand, to induce and amplify emotional states in patients; and on the other hand, to provide a system of visual and auditory representations of concepts, ideas, and memories that are meaningful to the patient. Therefore, the objective of EMMA’s World is to facilitate emotional change in people who have had traumatic, disturbing, and stressful experiences. The patient can visualize a virtual environment where a series of tools can be selected by the patient and the therapist. A series of emotional virtual objects and environments can be used and personalized so that they are meaningful to the patient. The objective is to obtain a physical representation of the personal meanings and emotions related to different patients’ problematic situations.

#### Scenarios, Special Effects, and Book of Life

The system includes five different pre-defined scenarios designed to reflect different emotions: a desert for anger, an island for relaxation, a threatening forest for anxiety, a snow-covered town for sadness, and a meadow for joy. In addition to making complete modifications in the virtual environment, the therapist and patient can graduate its intensity. Several special effects can be controlled: rainbow, rain, snow, storm, etc.; and it is also possible to modify the time of day (and its corresponding lighting), the music, and the sound. The patients can also select various symbols through a series of objects, videos, colors, and images. The *Book of Life* is another important tool in the system. In this book, the person can reflect on his or her emotional experience (with words, images, objects, music, etc.). All these elements are designed to activate and recall the traumatic or stressful events in order to achieve their emotional processing.

#### Control Interface

The therapist has a *control interface* that allows him or her to prepare in advance the elements that will be used in a specific session with a specific patient, and the therapist can also make changes in EMMA’s World in real time during the session. At each moment in the treatment, the therapist decides in which scenario to place the patient, depending on the emotion worked on in each session. These tools are not only used to help to induce a particular emotion, but also as a reflection of the emotions the patient may experience at any time during the therapy. Thus, the scenario and its different modulators can be adapted to the patient’s emotions at each point in the process, and the virtual environment ca be used to try to increase empathy with the patients. More detailed descriptions can be found in Rey et al. (2005), Botella et al. (2006), and Baños et al. (2009, 2011).

#### Software and Hardware

The following devices are necessary for the use EMMA’s World: two PCs[1], a large screen where the scenario is projected (5 mm thick, on an aluminum base measuring 2.50 × 1.80 metros), two projectors (SVGA Video projector, 1500 lumens), a wireless pad and a speakers system (Speakers 2.1. 40W). These devices were placed in a conditioned room to minimize the light in the room and achieve greater immersion in the system. One of the PCs is used to run the software, and the other PC is used by the therapist to make the changes in the virtual environment in real time, through a simple user interface. The software used to develop the application is *Brainstorm eStudio*. The PC that runs the virtual environment software has a graphic card with two connections; one of them is connected to a video projector that is used to project the image onto a methacrylate screen placed in the middle of the room. A wireless pad is placed on the other side of the room, and the patient can use it to interact and navigate in the virtual environment. The sound system includes several speakers distributed in the room.

### Treatment Protocols

In the case of the “traditional” condition, the treatment protocol was chosen that had the most empirical support for each of the three disorders. In the case of the “EMMA” condition, the same treatments protocols were adapted for application with the support of the VR system. Each treatment protocol is described below.

#### Post-traumatic Stress Disorder

In the case of PTSD, the “traditional” condition was based on the Foa and Rothbaum intervention (Foa and Kozak, 1986; Foa and Rothbaum, 1998), which included the following components: education, imaginal exposure, *in vivo* exposure, cognitive restructuring and strategies to cope with anxiety, such as breathing training, and finally, relapse prevention. Basic training in acceptance strategies was also added. In the EMMA condition, the same components were used, and the only difference was that the imaginal exposure was applied using VR.

#### Complicated Grief

The treatment protocol for the traditional component was created by our group partly following the guidelines and strategies recommended by Neimeyer (2000) and Neimeyer et al. (2002). It includes the following components: education, the *Book of Life*, restructuring the meaning of the loss, imaginal exposure, *in vivo* exposure, coping strategies to manage anxiety (such as slow breathing training), and relapse prevention. In addition, it includes a series of techniques for acceptance of loss, such as metaphors, a letter projecting into the future, etc. The only difference between the two conditions was that in the EMMA condition, the restructuring of the meaning was applied using the electronic version of the *Book of Life*. The EMMA condition is based on the same treatment protocol, but using new technologies.

#### Adjustment Disorders

The treatment protocol includes the following components: education, acceptance and elaboration of the negative events, and relapse prevention (Becker et al., 2007). It also includes positive psychology strategies that attempt to increase the human beings’ natural ability to resist adversity and use it to grow (Duckworth et al., 2005). Exercises are used to extract positive aspects of the situation experienced (Neimeyer, 2000; Neimeyer et al., 2002) and train the patient in coping with problems. However, D’Zurilla and Goldfried (1971) approach based on dimensions is not followed because the aim is for the patient to view the problems as necessary for growth and progress, and so Popper (1995) approach is used. The psychological treatment components were the same in both conditions, and the only difference was that the imaginal exposure was applied with VR support.

### Procedure

Individuals who showed interest in the treatment were interviewed to establish whether they met the inclusion and exclusion criteria. During this interview, the assessment and psychological treatment protocol was explained to the patients (psychological components included in the clinical protocol, number of sessions, number of assessment points, etc.), and they were told that this was a clinical trial with two experimental conditions, that both conditions were considered effective and that there was no risk identified in either of them. Once the patients agreed to participate in the study and signed the informed consent, all the patients were randomly assigned to one of the two experimental conditions: the “traditional condition” (*n* = 25) and the “EMMA condition” (*n* = 25) following the procedure explained above. After randomization, in the first therapy session the patients were informed what condition they had been assigned to.

The clinical assessment protocol was completed in two sessions before the treatment, and the same assessment protocol was applied again after the treatment.

The PTSD and CG treatment lasted approximately 9 weeks with one session per week (3 for the educational component, 5 for imaginal exposure/restructuring of the meaning of the loss, and 1 for relapse prevention). The AD treatment lasted approximately 6 weeks, with one session per week (1 for the educational component, 4 for exposure, and 1 for relapse prevention). Each exposure therapy session took approximately one and a half or 2 h. In all cases, for ethical reasons, people who needed it could extend the treatment for 3 weeks in the case of PTSD and CG, and 2 weeks for AD, until accomplishing all the behavioral objectives of the treatment.

Before starting the treatment, its rationale was explained in detail to the patients, and they filled out the “*Treatment Expectations Scale.”* Once they had finished the treatment, patients filled out the ‘*Treatment Satisfaction Scale’* and the *“Opinion Sheet on Treatment.”* In the case of the therapists, they were asked for their opinion about both treatments (“EMMA condition” and “traditional condition”) on two different “*Opinion Sheets on Treatment.’*

### Data Analysis

A mixed-methods approach using quantitative and qualitative methodologies was followed by the quantitative analyses were organized in two phases. First, *t*-statistics for testing the pre-test–post-test mean change were conducted separately for each treatment group, taking the six individual items of the satisfaction scale as the dependent variables, as well as the total score of the scale. Assuming a nominal significance level of 5%, probabilities were corrected following the Bonferroni method, so that statistical significance was reached only when the *p*-value was lower than 0.05/7 = 0.007. In addition, standardized pre-test–post-test mean change effect sizes (*d*) were calculated for each dependent variable, and a 95% confidence interval was calculated (Morris, 2008). Following Cohen (1988) *d* indices of about 0.5 and 0.8 were interpreted as indicating a moderate and large clinical magnitude, respectively. In a second phase, for each dependent variable a mixed ANOVA was applied, taking the treatment (Traditional vs. EMMA) as between-groups factor and the measurement occasion (pre-trial vs. post-trial) as the within-group one. Bonferroni adjustment was also applied in these ANOVAs. The *F*-test for the treatment x occasion interaction was complemented by calculating standardized pre-test–post-test mean change indices between the two groups for each dependent variable (*d*_change_), as well as a 95% confidence interval (Morris, 2008). The same Cohen (1988) guidelines were also applied to these effect sizes. All statistical analyses were carried out with SPSS 24.0.

Moreover, a qualitative methodology was applied following the principles of the Consensual Qualitative Research (CQR) approach (Hill et al., 1997, 2005; Hill, 2012). This qualitative tool is based on Grounded Theory (Glaser and Strauss, 1967), but specifically developed for clinical content. CQR involves asking the participants specific open-ended questions about a topic. Their responses are then coded into themes in a consensual manner by a group of researchers. The essential components of CQR (Hill et al., 2005) are the use of: (a) open-ended questions in semi-structured data collection techniques -usually in interviews-; (b) different judges throughout the data analysis process to provide multiple perspectives; (c) consensus strategies to arrive at judgments about the meaning of the data; (d) at least one auditor to check the work of the primary team, trying to minimize bias or the effects of groupthink; and (e) the generation of domains, core ideas, and cross-analyses in the data analysis. *The three steps for conducting CQR are developing and coding domains, constructing core ideas, and developing categories to describe consistencies across cases* (*cross analysis*). Consensus is a key part of the CQR method, which “relies on mutual respect, equal involvement, and shared power” (Hill et al., 1997). In this study, we use an adaptation of the CQR approach because we did not use interviews, but rather in-depth analysis of texts written by the participants to express their opinions about the treatments. Following the CQR guidelines, we had a primary team and an auditor, and in order to reach consensus, all the team members discussed disagreements and feelings at each step in the process. The primary team was composed of two members (one Master’s degree student and one Ph.D. student). The audit was performed by a third researcher (a Ph.D. student). First, the domains were established. The next step was the analysis of the core ideas, which were classified in the existing domains. Finally, a cross-analysis was carried out to establish the transversal categories. Each step was performed independently by the two researchers on the primary team. The auditor resolved the discrepancies at each step, except in the cross-analysis for the categories, which was independently carried out by the auditor. Once his/her categories had been generated, the auditor contrasted them with those generated by the primary team. The whole procedure was monitored by a Ph.D. full professor in clinical psychology.

## Results

The mean age of the sample was 31.28 years old (*SD*: 8.69) and ranged from 18 to 50 years. Most of the sample (70.0%) were female, and the remaining 30.0% were male; 60% single, 24% married, 2% widowers, and 14% divorced. With regard to the education level, 16% had an elementary level; 28% had a high school education level, and 56% had a university degree. Most of the sample (92%) did not have another Axis I diagnosis, and the rest (8%) did. Regarding other Axis II diagnoses, most of the sample (88%) did not have another Axis II diagnosis, and the rest (12%) presented Axis II co-morbidity.

### Patients’ Opinion: Quantitative Analysis

**Table 1** presents the results of the *t*-test comparison of pre-test–post-test means for the traditional therapy group. A first inspection of the data reveals averages for all items above seven points, including the total scale score, except for the item ‘unpleasantness’ in the pre-test, which is lower (mean = 5.81). A second result worthy of mention is that, in all items, and in the total score of the scale, higher means are observed in the post-test than in the pre-test, which indicates an improvement in the evaluation of the treatment by the patients. Thirdly, it is observed that, assuming a level of significance of 5%, statistically significant improvements were observed in items 2 and 3 and in the total score. However, if we apply the Bonferroni correction, none of these *t-*tests reaches statistical significance (*p* > 0.007). Fourth, and despite not achieving statistically significant results according to the Bonferroni correction, effect sizes, in terms of standardized mean change, are observed that can be considered clinically relevant according to the Cohen (1988) criterion for item 3 (recommend to others, *d* = 0.51), item 2 (*d* = 0.48) and for the total score (*d* = 0.46).

**Table 1:** Results of *t*-tests and effect sizes comparing expectation (pre-trial) and satisfaction (post-trial) of the patients in the traditional group.

Statement	Pre-trial *M* (*SD*)	Post-trial *M* (*SD*)	*t*	*p*	*d* (95%CI)
(1) Treatment logic	7.95 (1.91)	8.43 (1.16)	1.19	0.248	0.25 (−0.19, 0.69)
(2) Treatment satisfaction	7.76 (2.00)	8.86 (1.42)	2.29	0.033	0.48 (0.02, 0.94)
(3) Recommending to others	7.95 (1.96)	8.90 (1.37)	2.42	0.025	0.51 (0.04, 0.98)
(4) Usefulness for other disorders	7.57 (2.38)	7.95 (1.53)	0.77	0.451	0.16 (−0.28, 0.60)
(5) Usefulness for the patient	8.19 (1.99)	8.71 (1.42)	1.02	0.321	0.21 (−0.23, 0.65)
(6) Unpleasantness^∗^	5.81 (3.33)	7.14 (2.63)	1.68	0.109	0.35 (−0.10, 0.92)
Total score	7.54 (1.67)	8.33 (1.24)	2.18	0.041	0.46 (0.00, 0.92)

*M = mean. SD = standard deviation. t = t-test comparing the means for expectations (pre-trial) and satisfaction (post-trial); positive t-values indicated a larger mean in the post-trial than in the pre-trial. p = probability level associated to the t-test. d = standardized mean change effect size. 95%CI: 95% confidence limits for d. ^∗^Item 6 is answered in reverse, and so it has been recoded to follow the same scoring criteria as the other five items on the scale.*

**Table 2** presents the results of the *t*-test comparison of pre-test–post-test means for the EMMA therapy group. The data reveals averages for all items above 7.5 points, including the total scale score, except for the item ‘unpleasantness’ in the pre-test (mean = 5.91) and in the post-test (mean = 6.78). A second result worthy of mention is that, in all items, and in the total score of the scale, higher means are observed in the post-test than in the pre-test, which indicates an improvement in the evaluation of the treatment by the patients. Thirdly, it is observed that, assuming a level of significance of 5%, statistically significant improvements were observed in items 2, 3, 4 and 5, and in the total score. However, if we apply the Bonferroni correction, only item 3 (recommend to others, *p* = 0.002) and the total scale score (*p* = 0.002) reach statistical significance (*p* < 0.007). Fourth, we observe effect sizes, in terms of standardized mean change, that can be considered clinically very relevant according to Cohen (1988) criteria for the total score (*d* = 0.72), item 3 (recommend to others, *d* = 0.70), item 5 (usefulness for the patient, *d* = 0.50) and item 4 (usefulness for others, *d* = 0.47).

**Table 2:** Results of *t*-tests and effect sizes comparing expectation (pre-trial) and satisfaction (post-trial) of the patients in the EMMA group.

Statement	Pre-trial *M* (*SD*)	Post-trial *M* (*SD*)	*t*	*p*	*d* (95%CI)
(1) Treatment logic	8.39 (0.84)	8.70 (1.15)	1.19	0.245	0.24 (−0.18, 0.66)
(2) Treatment satisfaction	8.17 (1.70)	8.78 (1.20)	2.13	0.045	0.43 (−0.01, 0.87)
(3) Recommending to others	8.35 (1.43)	9.13 (1.10)	3.46	0.002	0.70 (0.23, 1.17)
(4) Usefulness for other disorders	7.83 (1.82)	8.70 (1.26)	2.33	0.030	0.47 (0.03, 0.91)
(5) Usefulness for the patient	8.04 (1.92)	8.78 (1.31)	2.49	0.021	0.50 (0.06, 0.94)
(6) Unpleasantness^∗^	5.91 (3.36)	6.78 (2.88)	1.17	0.255	0.24 (−0.18, 0.66)
Total score	7.78 (1.37)	8.48 (1.01)	3.58	0.002	0.72 (0.25, 1.19)

*M = mean. SD = standard deviation. t = t-test comparing the means for expectations (pre-trial) and satisfaction (post-trial); positive t-values indicated a larger mean in the post-trial than in the pre-trial. p = probability level associated to the t-test. d = standardized mean change effect size. 95%CI: 95% confidence limits for d. ^∗^Item 6 is answered in reverse, and so it has been recoded to follow the same scoring criteria as the other five items on the scale.*

**Table 3** presents the results of the mixed ANOVAs applied to the treatment condition (intergroup factor) and to the occasion of measurement (intra-group factor), taking as dependent variables the items and the total score of the scale. No statistically significant differences were observed between the two treatments. Statistically significant differences were observed for the average occasion in all items and in the total score, with the exception of item 1. However, applying the Bonferroni correction, only items 2 (*p* = 0.003) and 3 (recommend to others, *p* < 0.001) and the total score (*p* = 0.001) reached statistical significance (*p* < 0.007). Finally, and as a more relevant result, no significant effects of the interaction between the treatment and the measurement occasion were observed, indicating that the changes from pre-test to post-test were similar in the two treatments. In relation to the interaction, however, it is worth noting the obtaining of moderate-low magnitude effect sizes with items 4 (usefulness for other disorders, *d*_change_ = 0.31) and 5 (usefulness for other patients, *d*_change_ = 0.29) and with the total score (*d*_change_ = 0.26). The positive sign of these three effect size indices indicates a more pronounced pre-test–post-test change in the EMMA therapy group than in the traditional group.

**Table 3:** Results of the mixed ANOVAs taking treatment and occasion as inter-groups and within-group factors.

Statement	Treatment		Occasion		Interaction		*d*_change_ (95%CI)
	*F*	*p*	*F*	*p*	*F*	*p*	
(1) Treatment logic	1.22	0.276	2.81	0.101	0.14	0.714	−0.01 (−0.62, 0.60)
(2) Treatment satisfaction	0.18	0.675	9.75	0.003	0.79	0.378	−0.05 (−0.68, 0.58)
(3) Recommending to others	0.63	0.432	15.27	<0.001	0.15	0.704	0.19 (−0.47, 0.85)
(4) Usefulness for other disorders	1.26	0.267	4.15	0.048	0.63	0.430	0.31 (−0.31, 0.93)
(5) Usefulness for the patient	0.01	0.925	4.72	0.035	0.14	0.713	0.29 (−0.33, 0.91)
(6) Unpleasantness^∗^	0.03	0.865	4.10	0.049	0.18	0.672	−0.11 (−0.72, 0.50)
Total score	0.31	0.583	13.68	0.001	0.06	0.809	0.26 (−0.40, 0.92)

*F = F statistic for testing the significance of the treatment, the occasion, and the treatment × occasion interaction; all F-tests had 1 and 42 degrees of freedom. p = probability level associated to the F-test. d_change_ = standardized mean change between the two treatments (positive d_change_ values indicated a larger increase from expectations to satisfaction in EMMA than in TAU). 95%CI: 95% confidence limits for d_change_. ^∗^Item 6 is answered in reverse, and so it has been recoded to follow the same scoring criteria as the other five items on the scale.*

It should also be noted that mixed ANCOVAs were used to control the possible influence of covariates such as the degree of interference (pre-test and post-test), the consumption of medication and the history of the disorder, and that the results remained unchanged.

### Patients’ Opinion: Qualitative Analysis

This section presents the patients’ answers to the “Opinion Sheet on Treatment” after the intervention. **Tables 4, 5** show the domains, categories, and core ideas obtained from the patients’ opinion about the traditional and EMMA treatment conditions, respectively.

**Table 4:** Patient’s traditional condition.

Domains	Categories	Core ideas
*- Perception of change after treatment*	Positive change perceived (*general*)	“It was a very useful treatment for me. I am very grateful for everything”
*- Type of change*	Emotional change - Anxiety management *(typical)* - Cognitive change (variant) Coping - Relief (*typical*) - Resilience (*typical*)	“It helped me to manage the anxiety better, especially my pulse” Patient states that the treatment helped him in the way he thought Patient says she could talk normally about the issue with her grandmother “Overcome the problem helped me to feel very proud of myself”

**Table 5:** Patients: EMMA condition.

Domains	Categories	Core ideas
*- Perception of change after treatment*	Positive change perceived (*general*)	Patients say the treatment was very useful
*- Type of change*	Emotional change - Anxiety management (*typical*) - Acceptability (*variant*) Coping - Resilience (*typical*) - Relief (this category involves everything that means going back to the state before the stressful event, whereas the other category in fact increases after the stressor) Cognitive change (*typical)* Others (*rare*)	Patient states that she is less restless Patient states that she can express her emotions about the event without judging them Patient could fully cope with life Patient states that she is finally able to wipe him husband out of her mind Patient says a heavy load has been lifted Not as many memories come to my mind like before, and images come and go; they do not hurt; I’m not scared about keeping those images in my mind Improvements in sleeping
*- Perceptions about the treatment (protocol)*	- Re-experiencing the experience through the virtual system (*typical*) - Emotional processing (*typical*) - Treatment elements that have particularly helped them (*typical*)	Photographs helped me to place myself in the event and relive the accident “I found it easier to express emotions with the help of virtual environments and the devices that were included in the system” Patient stated that the photographs helped him to re-experience the situation
Strategies to improve the treatment	- New functionalities or evolutions of the system (variant) Usability aspects (*rare*)	Patient stated that it would be better to handle the system in an easier way Patient mentions that there should be more symbols

### Traditional Treatment

Regarding traditional treatment, only two domains were found (“Perception of change after treatment” and “Type of change”). It must be noted that all the patients had the opportunity to write whatever they wished about the treatment they received on an observation sheet.

In the domain “Perception of change after treatment,” all the patients’ comments were classified under the category “Positive change perceived.” In the domain “Type of change,” three categories were identified: “Emotional change” (typical), “Cognitive change” (variant) and “Coping,” which has two subcategories (“Relief” and “Resilience”). Based on the patient’s comments, it can be hypothesized that “Relief” is more typical of PTSD, as people start to feel like they did before the stressor, whereas “Resilience” is more present in cases of AD, where the patients easily feel that the situation was an opportunity for personal growth.

### EMMA Treatment

Regarding the EMMA treatment, and in contrast to the traditional condition, more domains were identified because the comments made by the patients were richer and more developed, leading to the identification of four domains “Perception of change after treatment,” “Type of change,” “Perceptions about the treatment,” and “Strategies to improve the treatment.”

The domain “Perception of change” was identified in all cases, which can be understood based on the success of the treatment delivery. The domain “Type of change” reflected the wide variety of changes that patients experienced, and they were classified in four categories: Emotional change (Anxiety management and Acceptance), Cognitive change, Coping (Resilience and Relief), and Other (including aspects such as sleeping better or improvements in interpersonal relationships).

The domain “Perception of the treatment” included three categories: “Re-experiencing the experience” (typical), “Emotional processing” (typical), and “Treatment elements particularly useful to them.” Finally, the last domain was “Strategies to improve the treatment,” which included two categories: “Usability aspects” (rare) and “New functionalities” (variant).

A few difficulties are described in the EMMA condition, but they do not include the necessary information to establish a domain with categories. An example is: “Sometimes I had difficulties in choosing the symbols; I would have liked it to be more personalized: images in real environments, instead of animated graphics.”

### Therapists’ Opinion: Qualitative Analysis

In this section, the therapists’ answers to the “Opinion Sheet on Treatment” about the two intervention protocols are analyzed. **Tables 6, 7** show the domains, categories, and core ideas obtained from the therapists’ opinions about the traditional and EMMA treatment conditions, respectively.

**Table 6:** Therapists: traditional condition.

Domains	Categories	Core ideas
*- Perception of change after treatment*	Positive change perceived (general)	Therapists say the treatment was very useful
*- Treatment features that have hindered the therapy process*	- Therapeutic techniques (*variant*) - Emotional processing *(typical)* - Specific elements (*rare*)	Therapists state that exposition *in vivo* is sometimes difficult to carry out Therapists say that there are patients that have difficulties in imagining the diverse situations Therapists state that exposure in imagination presents some problems, the application of this technique was quite hard to accomplish Therapists state that ideas expressed in words sometimes are not as intensely and realistically remembered as when using images
*-Treatment features that have helped them*	- Acceptability of the patients (*typical*) - Techniques/treatment components (*variant*) - Manualization of the treatments *(rare)*	Therapists state that patients feel understood and accept the treatment Therapists say that exposure in imagination and *in vivo* are very powerful and effective Therapists mention that having manualized treatment protocols makes it much easier when programming the sessions They are brief treatment protocols where an improvement is perceived from the very first sessions

**Table 7:** Therapists: EMMA condition.

Domains	Categories	Core ideas
*- Perception of change after treatment*	*Positive change perceived (general)*	Therapists say the treatment is a very useful treatment for patients
*- Treatment features that have helped them*	- Emotional processing *(general)* - Re-experiencing the experience through the virtual system *(typical)* - Specific elements or resources of the virtual system that have helped (*variant)* *- Acceptability of the treatment (variant)*	Therapists say that the use of symbols facilitates emotional expression Therapists express that, although re-experiencing can be painful, the system helped them to go through it Therapists state that using pictures and music selected by the patient has a powerful effect on the treatment Therapists say the system provides a safe environment
- Strategies	- *Usability (variant)* - *Technical features/resources (variant)*	Therapists state that the system should improve the ease of use of the treatment Therapists mention that it would be a good idea to incorporate the emotion thermometer in the system

### Traditional Treatment

Regarding the traditional treatment, three domains were identified: the “Perception of change after treatment,” “Treatment features that have hindered,” and “Treatment features that have helped them.” In the “Perception of change after treatment,” the only category that appeared in the analysis was “Positive change perceived.” This can be attributed to two aspects: on the one hand, the therapists were only asked to focus their comments on the treatments (protocols). On the other hand, the absence of other categories does not imply their inexistence because it is due to the small amount of information available for analysis. In this direction, all the answers in this domain about the perception of change after treatment were categorized as “Positive change perceived.”

In the “Treatment features that have hindered,” some negative comments are included. However, it must be noted that all the therapists took part in both conditions. Therefore, their answers tend to involve a comparison of the EMMA and the traditional conditions. Hence, the negative comments should not be considered intrinsically negative, but as comparisons to the EMMA condition. The therapists put special emphasis on the difficulty of “Emotional processing” in the traditional treatment. It has been classified as a typical category following the CQR principles. In this regard, it is quite linked to the category (variant) of “Therapeutic techniques,” which refers to therapists’ comments about the difficulties in applying techniques such as *in vivo* exposure and exposure in imagination. In addition, the category “Specific elements” (which is a rare category) highlights the scant use of elements such as symbols or metaphors. Therefore, the presence of all these categories may explain the potential of a virtual system like EMMA (this is developed in the EMMA condition analysis), which can deal with some aspects considered negative by the therapists.

In the “Treatment features that helped,” although many obstacles and barriers were described by the therapists, there is still a generally positive view of the protocol, as stated in the first domain. One of the aspects mentioned was the way the therapists thought the patients would accept the treatment. In this case, the category “Acceptability of the treatment” was classified as a variant, including aspects such as the flexibility of the manualization of the treatments.

### EMMA Treatment

In the EMMA condition, three domains were identified: “Perceptions of change after treatment,” “Treatment features that helped,” and “Strategies.”

In “Perceptions of change after treatment,” all the therapists reported a positive change in the patients after the treatment, leading to one single general category called “Positive change perceived,” as in the traditional condition. Therefore, the most interesting aspect lies in the differences in the specific processes the therapists said were helpful (particularly because all the therapists took part in both conditions, and their answers took their comparisons into consideration).

In the domain “Treatment features that helped,” and as in the traditional condition, the overall perception of change is also generally positive. Here, the “Emotional processing,” category, which is classified as general, is expressed as a particular positive aspect to be highlighted in the VR system. All the therapists except one emphasized the importance of “Emotional processing.” Likewise, the category “Re-experiencing the experience through the virtual system” (typical) was another important aspect described by the therapists that explains how the VR system can be better than the traditional treatment. Indeed, another category was identified that focuses on aspects the therapists described as improved in the *EMMA’s World* condition compared to the traditional treatment. Additionally, a variant category is called “Specific elements or resources of the virtual reality system that have helped,” such as “The book of life.” Last, but not least, there was a category called “Acceptability of the treatment,” which is coherent with the rest of the identified categories because it emphasizes that the VR system provides a safe environment.

There were also some negative features identified, but these aspects were not sufficient to build categories, due to the scarcity of the data collected. On this point, it must be noted that all the participants (both patients and therapists) were asked to answer the open questions about the treatment, but not all of them did so. Based on the data obtained, some of the potential categories on the negative aspects could be: (a) Patients’ reluctance to use the technology; (b) Rejection of the use of a VR system by the therapist due to the extra work that might be involved. Finally, in the domain “Strategies” to improve the system, there are two main categories: “Usability” and “Technical aspects and resources.” The therapists indicated that efforts were needed to make the technology very simple to use.

## Discussion

The objective of this study was to explore the opinions of patients and therapists about psychological treatment protocols supported by VR. Specifically, the application called *EMMA’s World*, a VR system designed for the treatment of stress-related disorders (PTSD, CG, and AD), was used. In particular, this study was aimed to analyze the usefulness of this VR system in increasing the efficiency (known as acceptability) of the psychological treatment protocols. Results indicate that *EMMA’s World* is well-accepted by both patients and therapists.

The expectations patients expressed about the treatments were very high in both conditions before starting the intervention, and patients’ opinion scores significantly improved after the treatment ended. No significant differences were found between the two conditions. However, effect sizes indicated a more pronounced pre-test–post-test change in the EMMA therapy group than in the traditional group.

The qualitative analysis showed that patients and therapists in both conditions perceived the treatment to be useful and efficacious. Nevertheless, the analysis of the patients in the EMMA condition provided much richer content. The patients perceived a positive change in general, identified a diverse range of types of changes and elements that helped them, and indicated that *EMMA’s World* strengthened motivation for the therapy and helped them to process the negative or stressful event they experienced throughout the treatment (“Photographs helped me to place myself in the event and relive the accident”; The “Book of life helped me to relive some good situations I experienced with my father”; “The background sound of the sea made me remember, and it helped me to bring to the surface feelings and sensations that I held and did not even recognize”).

In the case of therapists, because they participated in both conditions, the answers tended to be a comparison of the two conditions. All the therapists made positive comments about the usefulness of both treatments, but in the case of the traditional condition, they were more focused on efficacy (i.e., “Imaginal and *in vivo* exposure were very powerful tools that have been very effective”; “I find the treatment useful and effective”), whereas in the EMMA condition, the comments referred to how attractive it is for the patient (i.e., “The simple use of new technologies makes the treatment more attractive to the patient”; “The sessions using VR become much more pleasant, the patient is highly motivated, and the recovery is much faster”), emphasizing the flexibility (i.e., “Each session can be done in a very different way from the previous one, using other environments, other music, other symbols, and this is very attractive”) and the possibility of customizing it (i.e., “The possibility of customizing the scenario, including meaningful photographs, music, etc. for the patient, is very attractive”), in addition to helping the patients to better express their problems and emotions (i.e., “The patients told me that both the EMMA environment and the sounds or symbols helped them to express things that would have been very difficult to say in words”). Moreover, whereas some therapists identified some features that may be hindrances in the traditional condition (they recognized how difficult the imaginal technique was for some patients, and how hard the exposure technique was for the majority of them), they indicated that these limitations could be reduced with *EMMA’s World.* All of them found many positive features in the EMMA condition, especially in the exposure techniques and emotional processing, indicating that the VR system helped to foster motivation in patients while helping the therapist to apply the treatment. However, they also indicated that there is a need to improve the usability of the system (“I think anything that can facilitate the usability and friendliness of the system for patients should be improved, as well as the session preparations and the course on the sessions for therapists”). In conclusion, this analysis shows that VR can be a useful adjunct to treatment.

These results support those obtained in previous studies in this field. High rates of acceptance by the participants using VR have been observed, in comparison with *in vivo* exposure, for the treatment of phobias in subclinical populations (García-Palacios et al., 2001) and clinical populations (Botella et al., 2007; Quero et al., 2014). Similar results were obtained in the treatment of panic disorder with or without agoraphobia (Botella et al., 2007). In the case of the assessment and treatment of pain, patients have also shown high acceptance of the introduction of technology, and they have very positively appraised the use of technologies, highlighting that these tools could have a positive influence on the treatment outcomes (Spyridonis et al., 2013). In the same way, preliminary data in patients who suffer from stress-related disorders indicate high levels of satisfaction with the use of VR (Baños et al., 2009; Andreu-Mateu, 2011).

In this study, the fact that both patients and therapists rate the use of the VR system in a positive way is clearly encouraging. The incorporation of technology into the traditional treatment protocol has not *harmed* the patients’ expectations or opinions about it. Furthermore, in this study the treatment of choice was used as the control group, that is, as the “gold standard” from the perspective of the evidence-based treatment protocols (in the case of PTSD), or alternative treatments that had higher empirical support and were recommended in the specialized literature in the case of CG and AD. Quantitative analysis results indicate that the users’ (patients and therapists) assessment of VR is similar to what was obtained for *in vivo* exposure treatment, and qualitative analysis show that their opinions were even more positive. Including this type of VR-based element to assist the therapy could help to decrease the high percentage of therapists who are reluctant to use an effective technique like exposure.

This study has limitations. On the one hand, each patient only gave an opinion of the treatment applied to him or her, and so the opinion of a person who has first-hand information about both treatments remains to be explored. Only the therapists could assess both treatments because all of them delivered the treatments in both experimental conditions. On the other hand, the sample of therapists is small, and all of them received training to work with the VR system, but it is still necessary to explore the opinions of therapists who did not have any previous knowledge about the use of these technologies. However, the study by Segal et al. (2011) found that the therapists (*N* = 271) perceived that the benefits of using VR significantly exceeded the potential costs. In addition, the sample size of patients is not very large, and so future research should include a larger number of participants. Another limitation relates to the use of technology itself. Although our clinic is renowned for applying evidence-based psychological treatments, it is also well-known that some treatments are supported using technology. Therefore, although participants were invited to receive *psychological treatment* for their problems, perhaps more patients came in who did not reject the use of technology. Finally, it would also have been desirable to have stricter control over participants’ adherence to the treatment, but because the purpose of this study was to find out the patients’ opinion about the treatment they had received, only participants who finished the treatment in the previous studies were included in the present study.

Despite the encouraging results obtained in this study, there are still barriers to the routine use of VR in clinical practice (Riva, 2005; Segal et al., 2011). The first of these barriers refers to the economic costs of these systems, although, thankfully, costs have been declining rapidly in recent years, and progress is being made to implement these VR programs over the Internet, and even using smartphones. This, without a doubt, will simplify and reduce the costs of these programs. The second barrier is related to the possible technical difficulties that professional clinicians could have when using these systems, and the possible need for training to learn to use them. However, this topic always arises when new advances are made in treatments because clinicians have to be specifically trained to use them.

Many professionals are reluctant to introduce these tools in their clinical practice, or they feel threatened by the use of these technologies, perhaps because they do not understand the range of possibilities that these technologies can offer them. This problem is also linked to the lack of standardization in many of the *hardware* devices and *software.* To date, few available systems are interoperable, which makes it difficult to use them in different contexts from those for which they were developed. However, although this issue seems to be of great importance, it is gradually being overcome because the use of these technologies is being simplified as much as possible (user friendly systems), there are now training programs to teach these new strategies, and the advantages of these systems in clinicians’ daily work are clearly being demonstrated through data and empirical evidence. The expectation is that these types of systems will increasingly be developed (Newman et al., 2011).

Another possible barrier is the lack of standardized protocols shared by the scientific community, although this limitation is being corrected, and there are already some published protocols for the treatment of eating disorders (Riva et al., 2003), fear of flying (Klein, 1999), panic disorder (Vincelli et al., 2001; Botella et al., 2007), stress related disorders (Baños et al., 2009). Finally, there are ethical and safety issues (Durlach and Mayor, 1995; Botella et al., 2004). However, the data indicate that problems associated with VR are not frequent, and if they do appear, they are not serious, being limited to mild and transient discomfort and/or dizziness (Nichols and Patel, 2002).

We agree with Riva (2005), Riva and Repetto (2014), and Riva et al. (2015, 2016) that VR can become a substantial part of Clinical Psychology. For this reason, it is essential for all professionals to have a clear understanding of the opportunities that VR and other tools based on ICTs can provide in professional practice. To achieve this, on the one hand, it is necessary to carry out a proper dissemination and communication of the results that have already been obtained and, on the other, make progress in designing devices that are friendlier and easier to use at an affordable cost.

In sum, it seems that the use of technology in the field of Clinical Psychology is no longer a possibility. These tools have already shown their efficacy, and they are considered very promising and helpful elements in delivering psychological treatments (Clough and Casey, 2011; Turner and Casey, 2014; Freeman et al., 2017). Studies such as this one show that these tools are appreciated by users (both patients and therapists), and now it is time to incorporate them into daily clinical practice and show that they can be very useful for the variety of tasks that professionals should perform in the field of Clinical Psychology.

## Author Contributions

VG and RB made substantial contributions to the analysis and interpretation of the data, and they drafted the manuscript. VG, RB, and CB made substantial contributions to the conception and design of the study, the collection and interpretation of the data, and they revised the manuscript critically for important intellectual content. VG contributed to the collection, analysis and interpretation of the data, and drafted the manuscript. All authors provided final approval of the version to be published, and they agree to be accountable for all aspects of the work by ensuring that questions related to the accuracy or integrity of any part of the work are appropriately investigated and resolved.

## Conflict of Interest Statement

The authors declare that the research was conducted in the absence of any commercial or financial relationships that could be construed as a potential conflict of interest.
